# Vascular disrupting agent DMXAA enhances the antitumor effects generated by therapeutic HPV DNA vaccines

**DOI:** 10.1186/1423-0127-18-21

**Published:** 2011-03-08

**Authors:** Shiwen Peng, Archana Monie, Xiaowu Pang, Chien-Fu Hung, T-C Wu

**Affiliations:** 1 Department of Pathology, Johns Hopkins Medical Institutions, Baltimore, MD, USA; 2 Department of Obstetrics and Gynecology, Johns Hopkins Medical Institutions, Baltimore, MD, USA; 3 Department of Molecular Microbiology and Immunology, Johns Hopkins Medical Institutions, Baltimore, MD, USA; 4 Department of Oncology, Johns Hopkins Medical Institutions, Baltimore, MD, USA; 5 University College of Dentistry, Washington DC, USA

## Abstract

Antigen-specific immunotherapy using DNA vaccines has emerged as an attractive approach for the control of tumors. Another novel cancer therapy involves the employment of the vascular disrupting agent, 5,6-dimethylxanthenone-4-acetic acid (DMXAA). In the current study, we aimed to test the combination of DMXAA treatment with human papillomavirus type 16 (HPV-16) E7 DNA vaccination to enhance the antitumor effects and E7-specific CD8+ T cell immune responses in treated mice. We determined that treatment with DMXAA generates significant therapeutic effects against TC-1 tumors but does not enhance the antigen-specific immune responses in tumor bearing mice. We then found that combination of DMXAA treatment with E7 DNA vaccination generates potent antitumor effects and E7-specific CD8+ T cell immune responses in the splenocytes of tumor bearing mice. Furthermore, the DMXAA-mediated enhancement or suppression of E7-specific CD8+ T cell immune responses generated by CRT/E7 DNA vaccination was found to be dependent on the time of administration of DMXAA and was also applicable to other antigen-specific vaccines. In addition, we determined that inducible nitric oxide synthase (iNOS) plays a role in the immune suppression caused by DMXAA administration before DNA vaccination. Our study has significant implications for future clinical translation.

## Introduction

Advanced stage cancers are difficult to control using conventional therapies such as chemotherapy, surgery and radiation. Therefore, new innovative therapies are urgently required in order to combat the high mortality and morbidity associated with cancers. Antigen-specific immunotherapy has emerged as an attractive approach for the treatment of cancers since it has the ability to specifically eradicate systemic tumors and control metastases without damaging normal cells. DNA vaccination has become a potentially promising approach for antigen-specific immunotherapy due to its safety, stability and ease of preparation (for review, see [1,2]). We have previously developed several innovative strategies to enhance DNA vaccine potency by directly targeting the DNA into the dendritic cells (DCs) in vivo via gene gun as well as by modifying the properties of antigen-expressing DCs (for review see [3,4]).

One of the strategies to enhance DNA vaccine potency uses intracellular targeting strategies to enhance MHC class I/II antigen presentation and processing in DCs. Previously, we have studied the linkage of calreticulin (CRT), a Ca^2+^-binding protein located in the endoplasmic reticulum (ER) (for review, see [5]) to several antigens, including human papillomavirus type-16 (HPV-16) E7 [6,7], E6 [8], and nucleocapsid protein of severe acute respiratory syndrome (SARS) coronavirus [9]. Intradermal administration of CRT linked to any of these target antigens led to a significant increase in the antigen-specific CD8+ T cell immune responses and impressive antitumor effects. Thus, CRT has been shown to be highly potent in enhancing the antigen-specific immune responses and antitumor effects generated by DNA vaccination in several preclinical models.

Another novel cancer therapy involves the employment of the vascular disrupting agent, 5,6-dimethylxanthenone-4-acetic acid (DMXAA). Vascular disrupting agents are a new class of potential anticancer drugs that selectively destroy the established tumor vasculature and shutdown blood supply to solid tumors, causing extensive tumor cell necrosis (For reviews see [10,11]). DMXAA is a synthetic flavonoid that induces the production of local cytokines including TNFα. DMXAA has been shown to induce antitumor effects in animal models, especially in combination with established anticancer agents. It has demonstrated a good safety profile and has been shown to be promising in phase I clinical trials [12].

In the current study, we aimed to test the combination of DMXAA treatment with E7 DNA vaccination to enhance the antitumor effects and E7-specific CD8+ T cell immune responses in treated mice. We also aimed at exploring the appropriate regimen and the mechanism of action of this drug. The clinical implications of the current study are discussed.

## Materials and methods

### Mice

C57BL/6 mice (5- to 8-week-old) were purchased from the National Cancer Institute (Frederick, MD). 5-8 week-old inducible nitric oxide synthase deficient (iNOS^-/-^) and wild-type control C57BL/6 mice were purchased from Jackson Laboratories (Bar Harbor, ME). 5-8 week old TNFα-/- and wild-type control C57BL/6 mice were purchased from Taconic (Hudson, NY). All animals were maintained under specific-pathogen free conditions, and all procedures were performed according to approved protocols and in accordance with recommendations for the proper use and care of laboratory animals.

### Peptides, antibodies and regents

The H-2K^b ^restricted HPV-16 E6 peptide, YDFAFRDL (E6 aa50-57), and the H-2D^b ^restricted HPV-16 E7 peptide, RAHYNIVTF (E7 aa49-57) were synthesized by Macromolecular Resources (Denver, CO) at a purity of ≥70%. FITC-conjugated rat anti-mouse CD4, CD8, IFN-γ and PE-conjugated anti-mouse CD8 antibodies were purchased from BD Pharmingen (BD Pharmingen, San Diego, CA). 5,6-dimethylxanthenone-4-acetic acid (DMXAA) was purchased from Sigma (St. Louis, MO). DMXAA was dissolved in 5% sodium bicarbonate, and injected intraperitoneally (i.p.) at a dose of 20 mg/kg of body weight.

### Cells

HPV-16 E6 and E7-expressing TC-1 tumor cells were generated as previously described [13] and was grown in RPMI 1640 medium containing 10% fetal bovine serum, 2 mM glutamine, 1 mM sodium pyruvate, 100 IU/ml penicillin, 100 μg/ml streptomycin, 100 μM non-essential amino acids and 0.4 mg/ml of G418.

### Vaccines

The generation of HPV-16 E7-expressing plasmid (pcDNA3-CRT/E7), E6-expressing plasmid (pcDNA3-CRT/E6) [8], PADRE-expressing plasmid (pcDNA3-IiPADRE) [14], and vaccinia virus encoding HPV-16 E7 (SigE7LAMP1) [15], has been described previously.

### Mouse tumor challenge model

C57BL/6 mice (five per group) were injected with 1 × 10^5 ^TC-1 tumor cells subcutaneously at the flank site in 100 μL PBS. Tumors were measured twice a week. Tumor volume was estimated using the formula 3.14 × [largest diameter × (perpendicular diameter)^2^]/6.

### Vaccination

Preparation of DNA-coated gold particles and gene gun particle-mediated DNA vaccination was performed using a helium-driven gene gun (BioRad Laboratories Inc., Hercules, CA) according to a protocol described previously. Gold particles coated with pcDNA3 encoding HPV-16 E6 or HPV-16 E7 or PADRE were delivered to the shaved abdominal region of mice using a helium-driven gene gun with a discharge pressure of 400 psi. Mice were immunized with 2 μg of the various DNA vaccines and received boosts with the same regimen as indicated in the figure legends. For vaccinia encoding SigE7LAMP1 vaccination, 1 × 10^7 ^pfu viruses were injected intraperitoneally in 100 μl volume. Splenocytes were harvested 1 week after the last vaccination.

### Intracellular cytokine staining and flow cytometry analysis

Before intracellular cytokine staining, pooled splenocytes from each vaccination group were incubated for 20 hours with 1 μg/ml of the HPV-16 E6 aa50-57 peptide, or HPV-16 E7aa49-57 peptide, or PADRE peptide at the presence of GolgiPlug (BD Pharmingen, San Diego, CA). The stimulated splenocytes were then washed once with FACScan buffer and stained with PE-conjugated monoclonal rat antimouse CD8a (clone 53.6.7). Cells were subjected to intracellular cytokine staining using the Cytofix/Cytoperm kit according to the manufacturer's instruction (BD Pharmingen, San Diego, CA). Intracellular IFN-γ was stained with FITC-conjugated rat antimouse IFN-γ. Flow cytometry analysis was performed using FACSCalibur with CELLQuest software (BD biosciences, Mountain View, CA).

### Detection of T cell apoptosis

C57BL/6 mice were treated with DMXAA at 20 mg/kg via i.p. injection. 48 hours later, splenocytes were harvested and apoptosis of T cells were analyzed by staining splenocytes with annexin V staining kit from BD Pharmingen according to the protocol provided by the manufacturer.

### Bio-Plex cytokine assay

5~8 week-old C57BL/6 mice were vaccinated with 2 μg of pcDNA3-CRT/E7 DNA via gene gun delivery. 3 days after the vaccination, the mice were treated with either 20 mg/kg of DMXAA or buffer via i.p. injection. Mouse serum was collected 5 hours later and stored at -80°C until assay. Mouse cytokines were analyzed using Bio-Plex Pro Mouse Cytokine 23-plex Assay from Bio-Rad according to manufacturer's protocol. Each sample was assayed in duplicate.

### Statistical analysis

Data expressed as means ± standard deviations (SD) are representative of at least two different experiments. Comparisons between individual data points were made by 2-tailed Student's *t *test. A *p *value of less than 0.05 was considered significant.

## Results

### Treatment with DMXAA generates significant therapeutic effects against TC-1 tumors but does not enhance the antigen-specific immune responses in tumor bearing mice

**Figure 1:**
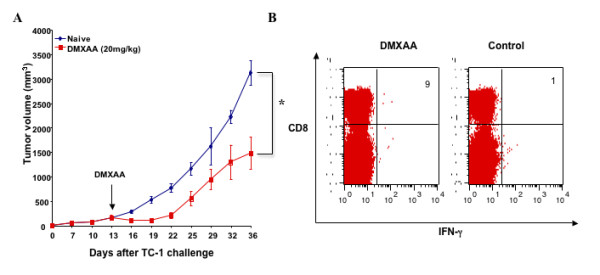
**Characterization of antitumor effects and E7-specific CD8+ T cell immune responses in TC-1 tumor-bearing mice treated with DMXAA**. 5-8 weeks old C57BL/6 mice (5 per group) were challenged with 1 × 10^5 ^TC-1 cells subcutaneously. Mice were treated with a single dose of DMXAA given at day 13 after tumor challenge via i.p. injection. Tumor volume was monitored with calipers twice a week. One week after DMXAA treatment, splenocytes from tumor-bearing mice were harvested and characterized for E7-specific CD8^+ ^T cells using intracellular IFN-*γ *staining followed by flow cytometry analysis. **A) **Line graph depicting the tumor volume in TC-1 tumor bearing mice treated with or without DMXAA (mean+ s.e.) **B) **Representative data of intracellular cytokine staining followed by flow cytometry analysis showing the number of E7-specific IFNγ+ CD8+ T cells in after DMXAA treatment. The data shown here are from one representative experiment of two performed.

To determine the antitumor effects of treatment with DMXAA, we first challenged groups of C57BL/6 mice (5 per group) with TC-1 tumor cells and treated them with a single dose of DMXAA which was administered on day 13 after tumor challenge via i.p. injection and monitored the tumor size over time. As shown in Figure 1A, tumor bearing mice treated with DMXAA showed significantly lower tumor volumes over time compared to tumor bearing mice without DMXAA treatment (* p < 0.05). We also characterized the E7-specific CD8^+ ^T cell immune responses in these mice. One week after DMXAA treatment, splenocytes from tumor-bearing mice were harvested and characterized for E7-specific CD8^+ ^T cells using intracellular IFN-*γ *staining followed by flow cytometry analysis. However, as shown in Figure 1B, we found that mice treated with DMXAA were not capable of significantly enhancing the E7-specific CD8+ T cell immune responses compared to mice without DMXAA treatment. Taken together, our data indicate that treatment with DMXAA generates significant therapeutic effects against TC-1 tumors but does not enhance the antigen-specific immune responses in tumor bearing mice.

### Combination of DMXAA treatment with E7 DNA vaccination generates potent antitumor effects and E7-specific CD8+ T cell immune responses in the splenocytes of tumor-bearing mice

**Figure 2:**
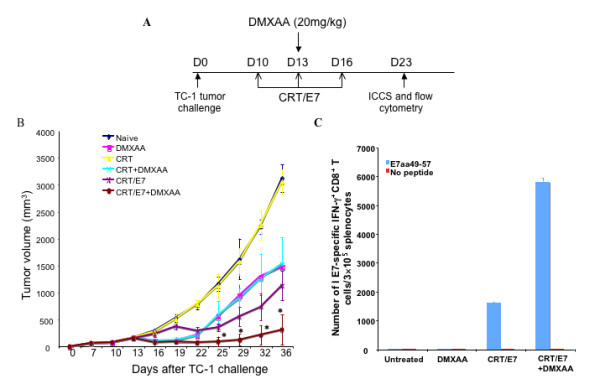
**Characterization of antitumor effects and E7-specific CD8+ T cell immune responses in tumor-bearing mice treated with HPV-16 E7 DNA vaccine in combination with DMXAA**. **(A) **Schematic diagram of the immunization regimen of the CRT/E7 DNA vaccine and/or DMXAA. 5-8 weeks old C57BL/6 mice (5 per group) were challenged with 1 × 10^5 ^TC-1 tumor cells subcutaneously, and were vaccinated with pcDNA3-CRT/E7 DNA vaccine or control vectors, and either treated with DMXAA or left untreated as indicated. Tumor volume was monitored with calipers twice a week. One week after last vaccination, splenocytes from tumor-bearing mice were harvested and characterized for E7-specific CD8^+ ^T cells using intracellular IFN-*γ *staining followed by flow cytometry analysis. **A) **Line graph depicting the tumor volume in TC-1 tumor bearing mice treated with the various regimens (mean+ s.e.) **B) **Bar graph depicting the number of E7-specific IFNγ+ CD8+ T cells per 3 × 10^5 ^splenocytes ± SEM following DNA vaccination +/- DMXAA treatment. The data shown here are from one representative experiment of two performed.

In order to determine the therapeutic antitumor effects and E7-specific CD8+ T cell immune response in TC-1 tumor-bearing mice treated with DMXAA combined with CRT/E7 DNA vaccination, we first challenged groups of C57BL/6 mice (5 per group) with TC-1 tumor cells and then treated them with CRT/E7 DNA vaccine with or without DMXAA as illustrated in Figure 2A. Seven days after the last vaccination, we harvested splenocytes from vaccinated mice and characterized them for the presence of E7-specific CD8^+ ^T cells using intracellular cytokine staining for IFN-γ followed by flow cytometry analysis. As shown in Figure 2B, tumor-bearing mice that were treated with CRT/E7 DNA vaccine in combination with DMXAA generated the best therapeutic antitumor effects compared to mice treated with any other regimens (* p < 0.05). Furthermore, mice treated with DNA vaccine in combination with DMXAA also generated the highest number of E7-specific CD8^+ ^T cells compared to mice treated with any of the other regimens. Thus, our results suggest that treatment of tumor-bearing mice with DMXAA enhances the systemic E7-specific CD8+ T cell immune responses and antitumor effects generated by CRT/E7 DNA vaccination.

### **The DMXAA-mediated enhancement of E7-specific CD8+ T cell immune responses generated by CRT/E7 DNA vaccination is dependent on the time of administration of DMXAA**

**Figure 3:**
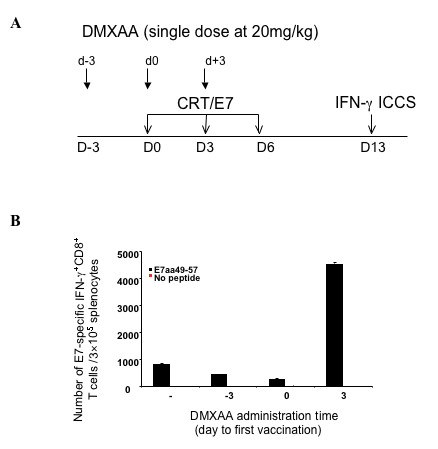
**Characterization of the E7-specific CD8+ T cell immune responses in naïve mice treated with HPV-16 E7 DNA vaccine in combination with DMXAA administered at different time points**. **(A) **Schematic diagram of the immunization regimen of the CRT/E7 DNA vaccine and/or DMXAA administered at different time points, either 3 days before the first vaccination (d-3), simultaneously (d0) or 3 days after the first vaccination (d+3). 5-8 weeks old C57BL/6 mice were vaccinated with pcDNA3-CRT/E7 DNA vaccine via gene gun delivery and treated with DMXAA as indicated in **Figure 3A**. One week after last vaccination, splenocytes from mice were harvested and characterized for E7-specific CD8^+ ^T cells using intracellular IFN-*γ *staining followed by flow cytometry analysis. (**B) **Bar graph depicting the number of E7-specific IFNγ+ CD8+ T cells per 3 × 10^5 ^splenocytes ± SEM following DNA vaccination +/- DMXAA treatment. The data shown here are from one representative experiment of two performed.

In order to determine the optimal regimen for enhancing the antigen-specific CD8+ T cell immune responses generated by CRT/E7 DNA vaccine using DMXAA, C57BL/6 mice (5 per group) were vaccinated with CRT/E7 DNA vaccine three times at 3 day intervals via gene gun delivery and treated with DMXAA at 3 days before the first vaccination (-3), simultaneously (0) or 3 days after the first vaccination (+3) as indicated in Figure 3A. Vaccinated mice without DMXAA treatment were used as controls. Seven days after the last vaccination, splenocytes were harvested from vaccinated mice and characterized for the presence of E7-specific CD8^+ ^T cells using intracellular cytokine staining for IFN-γ followed by flow cytometry analysis. As shown in Figure 3B, vaccinated mice treated with DMXAA 3 days after vaccination generated the best E7-specific CD8+ T cell immune responses compared to any of the other regimens. Furthermore, we observed that vaccinated mice treated with DMXAA at the time of vaccination or 3 days before the first vaccination generated suppressed E7-specific CD8+ T cell immune responses compared to vaccinated mice without DMXAA treatment. Thus, our data indicate that administration of DMXAA 3 days after the first CRT/E7 DNA vaccination generates significantly enhanced E7-specific CD8+ T cell immune responses in tumor-bearing mice.

**Figure 4:**
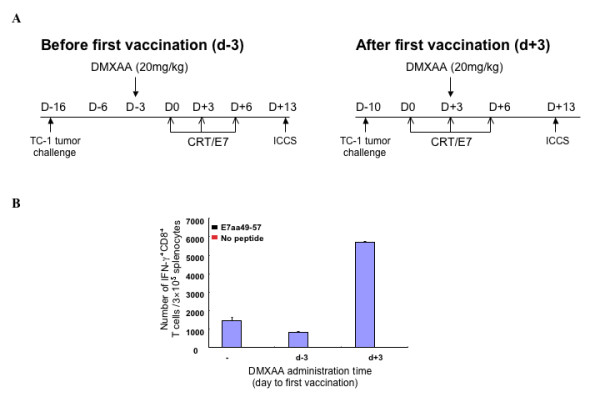
**Characterization of the E7-specific CD8+ T cell immune responses in tumor-bearing mice treated with HPV-16 E7 DNA vaccine in combination with DMXAA administered at different time points**. **(A) **Schematic diagram of the immunization regimen of the CRT/E7 DNA vaccine and/or DMXAA administered at different time points, either 3 days before the first vaccination (d-3) or 3 days after the first vaccination (d+3). 5-8 weeks old C57BL/6 mice were challenged with 1 × 10^5 ^TC-1 tumor cells subcutaneously, vaccinated with pcDNA3-CRT/E7 DNA vaccine via gene gun delivery and treated with DMXAA as indicated in **Figure 4A**. One week after last vaccination, splenocytes from tumor-bearing mice were harvested and characterized for E7-specific CD8^+ ^T cells using intracellular IFN-*γ *staining followed by flow cytometry analysis. (**B) **Bar graph depicting the number of E7-specific IFNγ+ CD8+ T cells per 3 × 10^5 ^splenocytes ± SEM following DNA vaccination +/- DMXAA treatment. The data shown here are from one representative experiment of two performed.

In order to determine if the observed phenomenon is also applicable to tumor-bearing mice, C57BL/6 mice (5 per group) were challenged with TC-1 tumor cells subcutaneously, vaccinated with pcDNA3-CRT/E7 DNA vaccine via gene gun delivery, and treated with DMXAA either before the first vaccination (d-3) or after the first vaccination (d+3) as indicated in Figure 4A. One week after last vaccination, splenocytes from tumor-bearing mice were harvested and characterized for E7-specific CD8^+ ^T cells using intracellular IFN-*γ *staining followed by flow cytometry analysis. As shown in Figure 4B, tumor-bearing mice treated with DMXAA 3 days after the first vaccination (d+3) generated significantly higher E7-specific CD8+ T cell immune responses compared to tumor-bearing mice treated with DMXAA before vaccination (d-3) (p < 0.05). We also observed that vaccinated tumor-bearing mice treated with DMXAA at the time of vaccination or 3 days before vaccination generated suppressed E7-specific CD8+ T cell immune responses compared to vaccinated mice without DMXAA treatment.

Furthermore, tumor-bearing mice treated with DMXAA 3 days after the first vaccination (d+3) generated a significantly increased number of activated dendritic cells compared to the control. In addition, treatment with DMXAA also led to increased expression of co-stimulatory markers for DC activation compared to the control (see Additional File 1; Figure S1). The increased number and function of DCs contribute to the enhanced processing and presentation of E7 antigen to the generation of E7-specific CD8+ T cells in treated mice. Taken together, our data indicate that the timing of administration of DMXAA significantly influences the E7-specific CD8+ T cell immune responses in treated mice.

### The DMXAA-mediated enhancement of antigen-specific T cell-mediated immune responses generated by vaccination is also applicable to other antigen-specific vaccines

**Figure 5:**
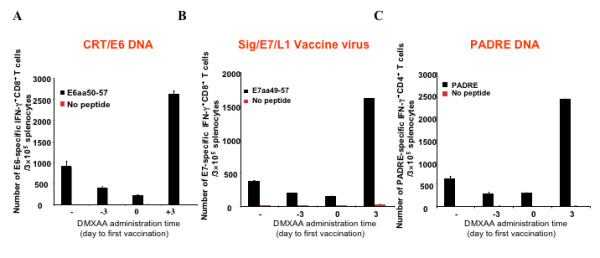
**Characterization of the antigen-specific CD8+ T cell immune responses in mice treated with various vaccines in combination with DMXAA administered at different time points**. 5-8 weeks old C57BL/6 mice were vaccinated with CRT/E6 DNA via gene gun, Sig/E7/L1 vaccinia virus intraperitoneally or PADRE DNA vaccine via gene gun delivery and treated with DMXAA at 3 days before vaccination (-3), simultaneously (0) or 3 days after vaccination (+3) as indicated in Figure 3A. One week after last vaccination, splenocytes from mice were harvested and characterized for **(A) **HPV-16 E6aa50-57-specific CD8^+ ^T cell responses, **(B) **HPV-16 E7aa49-57-specific CD8^+ ^T cell responses, or **(C) **PADRE-specific CD4^+ ^T cell responses using intracellular IFN-*γ *staining followed by flow cytometry analysis. **A & B**. Bar graph depicting the number of antigen-specific IFNγ+ CD8+ T cells per 3 × 10^5 ^splenocytes ± SEM following vaccination +/- DMXAA treatment. **C**. Bar graph depicting the number of antigen-specific IFNγ+ CD4+ T cells per 3 × 10^5 ^splenocytes ± SEM following vaccination +/- DMXAA treatment. The data shown here are from one representative experiment of two performed.

In order to determine if the observed enhancement of HPV DNA vaccine-induced antigen-specific immune responses by DMXAA is also applicable to other antigen-specific vaccines, C57BL/6 mice (5 per group) were vaccinated with CRT/E6 DNA or Sig/E7/L1 vaccinia virus or PADRE DNA vaccine via gene gun delivery and treated with DMXAA at 3 days before vaccination (-3), simultaneously (0) or 3 days after vaccination (+3) as indicated in Figure 3A. One week after last vaccination, splenocytes from mice were harvested and characterized for antigen-specific T cell immune responses using intracellular IFN-*γ *staining followed by flow cytometry analysis. As shown in Figure 5, mice vaccinated with the 3 different vaccines (CRT/E6 DNA or Sig/E7/L1 vaccinia virus or PADRE DNA) and treated with DMXAA 3 days after the first vaccination all generated the best antigen-specific T cell immune responses (**(A) **HPV-16 E6-specific CD8^+ ^T cell responses, **(B) **HPV-16 E7-specific CD8^+ ^T cell responses, and **(C) **PADRE-specific CD4^+ ^T cell immune responses) compared to any of the other regimens. Thus, our data indicate that administration of DMXAA three days after the first vaccination is capable of enhancing antigen-specific immune responses in different vaccination systems.

In order to determine if additional doses of DMXAA following the first vaccination would further enhance the immune responses generated in vaccinated mice, C57BL/6 mice (5 per group) were vaccinated with pcDNA3-CRT/E7 DNA vaccine via gene gun delivery and treated with either one dose or two doses of DMXAA as indicated in Additional File 2; Figure S2A. One week after last vaccination, splenocytes from mice were harvested and characterized for E7-specific CD8^+ ^T cells using intracellular IFN-*γ *staining followed by flow cytometry analysis. As shown in Additional File 2; Figure S2B and **C**, vaccinated mice treated with two doses of DMXAA after vaccination generated significantly better E7-specific CD8+ T cell immune responses compared to vaccinated mice treated with one dose of DMXAA. Thus, our data indicate that administration of two doses of DMXAA after the first CRT/E7 DNA vaccination generates significantly better E7-specific CD8+ T cell immune responses in vaccinated mice compared to administration of one dose of DMXAA.

### Co-administration of DMXAA with CRT/E7 DNA vaccine generates long term E7-specific memory CD8+ T cell immune responses in vaccinated mice

**Figure 6:**
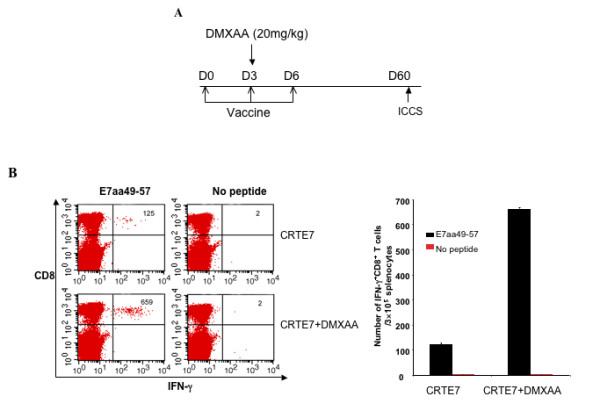
**Characterization of the E7-specific memory CD8+ T cell immune responses in mice treated with HPV-16 E7 DNA vaccine in combination with DMXAA**. **(A) **Schematic diagram of the immunization regimen of the CRT/E7 DNA vaccine and/or DMXAA. 5-8 weeks old C57BL/6 mice were vaccinated with pcDNA3-CRT/E7 DNA vaccine via gene gun delivery at day 0, 3 and 6 and DMXAA (20 mg/kg) was administered on day 3 as depicted in Figure 6A. Sixty days after the first vaccination, splenocytes were harvested and characterized for E7-specific CD8^+ ^T cells using intracellular IFN-*γ *staining followed by flow cytometry analysis. **(B) **Representative data of intracellular cytokine staining followed by flow cytometry analysis showing the number of E7-specific IFNγ+ CD8+ T cells in after DNA vaccination +/- DMXAA treatment. **(C) **Bar graph depicting the number of E7-specific IFNγ+ CD8+ T cells per 3 × 10^5 ^splenocytes ± SEM following DNA vaccination +/- DMXAA treatment. The data shown here are from one representative experiment of two performed.

In order to determine the long-term memory T cell immune responses generated by CRT/E7 DNA vaccination with or without treatment with DMXAA, C57BL/6 mice (5 per group) were vaccinated with CRT/E7 DNA vaccine three times with 3 day intervals via gene gun delivery and treated with DMXAA at 3 days after vaccination as indicated in Figure 6A. Sixty days after the last treatment, we harvested splenocytes from vaccinated mice and characterized them for the presence of E7-specific CD8^+ ^T cells using intracellular cytokine staining for IFN-γ followed by flow cytometry analysis. As shown in Figure 6B, vaccinated mice treated with DMXAA 3 days after the first vaccination generated significantly better E7-specific CD8+ memory T cell immune responses compared to vaccination without DMXAA treatment. Thus, our data indicate that administration of DMXAA 3 days after the first CRT/E7 DNA vaccination enhances the E7-specific CD8+ memory T cell immune responses in vaccinated mice.

### Co-administration of DMXAA with DNA vaccine leads to elevated levels of inflammatory cytokines in the serum of treated mice

**Figure 7:**
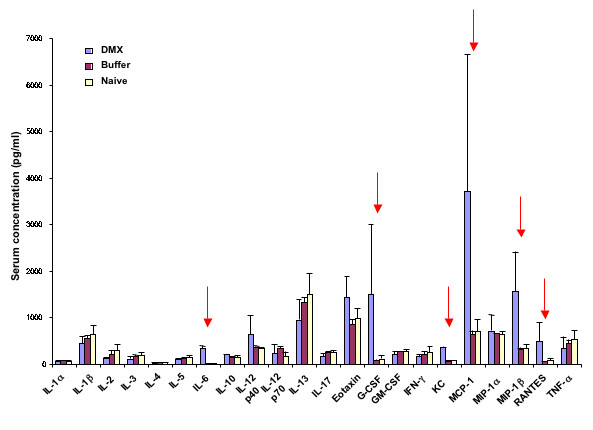
**Multiplex analysis to determine the serum cytokine concentration from CRT/E7 DNA vaccinated mice either treated with DMXAA**. 5-8 week-old C57BL/6 mice were vaccinated with 2 μg of pcDNA3-CRT/E7 DNA via gene gun delivery. 3 days after the vaccination, the mice were treated with either 20 mg/kg of DMXAA or buffer via i.p. injection. Mouse serum was collected 5 hours later and stored at -80°C until assay. Mouse cytokines were analyzed using Bio-Plex Pro Mouse Cytokine 23-plex Assay from Bio-Rad according to manufacturer's protocol. Each sample was assayed in duplicate. The data are expressed as means ± SD.

In order to determine if co-administration of DMXAA with DNA vaccination will influence the cytokine level in the serum of mice with observed immune enhancement, we characterized the serum cytokine concentration from vaccinated mice treated with DMXAA 3 days after the first vaccination (see Figure 6A) using multiplex analysis. As shown in Figure 7, the cytokines IL-6, G-CSF, KC, MIP-1β, MCP-1 and RANTES were found to be elevated in vaccinated mice treated with DMXAA compared to vaccinated mice without DMXAA treatment. These cytokines may potentially play a role in the enhancement of antigen-specific T cell immune responses caused by co-administration of DMXAA with the DNA vaccine.

### **iNOS plays a role in the immune suppression caused by DMXAA administration at the time of the first DNA vaccination**

In order to determine the mechanism by which DMXAA leads to suppressed antigen-specific CD8+ T cell immune responses when administered before or at the time of the first DNA vaccination, we characterized the apoptotic cell death of CD4+ and CD8+ T cells in the splenocytes derived from mice treated with DMXAA. C57BL/6 mice (5 per group) were treated with DMXAA at 20 mg/kg via i.p. injection. 48 hours later, splenocytes were harvested and apoptosis of CD4+ and CD8+ T cells were analyzed by annexin V staining. There was no significant difference in the levels of apoptotic cell death in the CD4+ or CD8+ T cells among splenocytes from mice treated with DMXAA compared to those from the control mice (see Additional File 3; Figure S3). Thus, our data suggest that the mechanism by which DMXAA leads to suppressed antigen-specific immune responses is not through T cell apoptosis.

**Figure 8:**
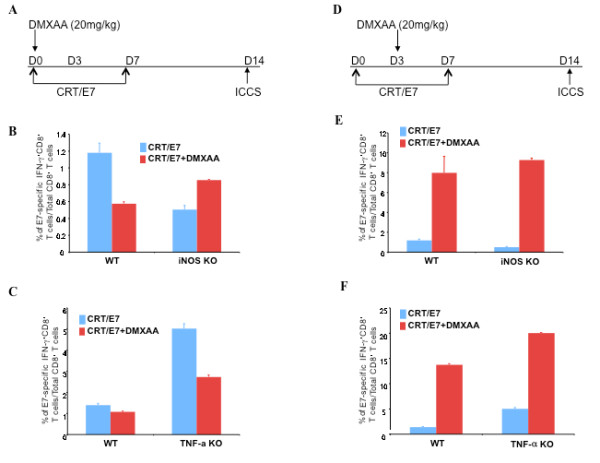
**Characterization of the E7-specific CD8+ T cell immune responses in iNOS and TNF-α knockout mice treated with HPV-16 E7 DNA vaccine in combination with DMXAA**. **A & B**. Schematic diagram of the immunization regimen of the CRT/E7 DNA vaccine and DMXAA administered at different time points. 5-8 weeks old wild-type or iNOS deficient (middle panel), or TNF-α deficient (bottom panel) C57BL/6 mice were vaccinated with pcDNA3-CRT/E7 via gene gun delivery at day 0, and boosted once at day 7. The mice were treated with DMXAA (20 mg/kg) via i.p. injection either at day 0 **(A) **or day 3 **(D) **of the first vaccination. Splenocytes were harvested 7 days after last vaccination, and HPV-16 E7aa49-57-specific-CD8+ T cell responses were analyzed by intracellular IFN-γ staining using flow cytometry. **B & C**. Bar graphs depicting the number of E7-specific IFNγ+ CD8+ T cells per 3 × 10^5 ^splenocytes in WT, iNOS-/-mice **(B) **or TNF-α-/- mice **(D) **± SEM following DNA vaccination and DMXAA treatment on D0. **E & F**. Bar graphs depicting the number of E7-specific IFNγ+ CD8+ T cells per 3 × 10^5 ^splenocytes in WT, iNOS-/-mice **(E) **or TNF-α-/- mice **(F) **± SEM following DNA vaccination and DMXAA treatment on D3.The data shown here are from one representative experiment of two performed.

It has been shown that mice treated with DMXAA have been shown to induce iNOS production as well as TNFα in tumors [16]. Furthermore, iNOS and TNFα has been implicated in playing an important role in antitumor immunity (for reviews, see [17-19]. Thus, in order to further explore the mechanism of action of DMXAA related to iNOS and TNFα, we have used iNOS-/- mice or TNFα-/- mice as well as C57BL/6 WT mice (5 per group) for our study. These mice were vaccinated with CRT/E7 DNA vaccine via gene gun delivery and treated with DMXAA either at the time of first vaccination on D0 or 3 days after the first vaccination on D3 as indicated in Figure 8A and 8D. One week after last vaccination, splenocytes from vaccinated mice were harvested and characterized for E7-specific CD8^+ ^T cells using intracellular IFN-*γ *staining followed by flow cytometry analysis. As shown in Figure 8B, while DMXAA led to the suppression of E7-specific CD8+ T cell immune responses in CRT/E7 vaccinated WT mice when administered on D0, DMXAA did not suppress the E7-specific CD8+ T cell immune responses in CRT/E7 vaccinated iNOS-/- mice. This indicates that iNOS is a major factor in the immunosuppression mediated by DMXAA when administered at the time of the first DNA vaccination. On the other hand, vaccinated TNFα-/- mice treated with DMXAA administered on D0 suppressed the E7-specific CD8+ T cell immune responses similar to wild-type mice (see Figure 8C). We also found that vaccinated iNOS-/- mice or TNFα-/- mice treated with DMXAA on D3 led to enhancement E7-specific CD8+ T cell immune responses similar to wild-type mice (Figure 8E and 8F). Thus, our data indicate that iNOS, but not TNFα contribute to the observed immune suppression caused by DMXAA administration at the time of the first DNA vaccination.

## Discussion

In the current study, we determined that treatment with DMXAA generates significant therapeutic effects against TC-1 tumors but does not enhance the antigen-specific immune responses in tumor bearing mice. We further found that combination of DMXAA treatment with therapeutic HPV DNA vaccination generates potent antitumor effects and E7-specific CD8+ T cell immune responses in tumor bearing mice. Furthermore, the DMXAA-mediated enhancement or suppression of E7-specific CD8+ T cell immune responses generated by CRT/E7 DNA vaccination was found to be dependent on the time of administration of DMXAA and was also applicable to other antigen-specific vaccines. In addition, we determined that iNOS plays a role in the immune suppression caused by DMXAA administration before DNA vaccination. Our data are consistent with a recent observation using E7 peptide-based vaccines in an E7-expressing cervicovaginal tumor model [20].

In our study, we observed that treatment of tumor-bearing mice with DMXAA alone leads to therapeutic antitumor effects without generating antigen-specific immune responses (Figure 1). This may be due to the fact that as a vascular disrupting agent, DMXAA has been shown to exert antitumor effects by non antigen-specific mechanisms such as selectively destroying the established tumor vasculature and shutting down blood supply to solid tumors, causing extensive tumor cell necrosis [10,11]. The release of tumor antigen caused by DMXAA treatment may not be sufficient to generate detectable antigen-specific immune responses. Thus, while DMXAA treatment alone in TC-1 tumor-bearing mice failed to lead to appreciable E7 antigen-specific immune responses, the vaccination with CRT/E7 vaccine can lead to increased number of E7-specific CD8+ T cell precursors in tumor-bearing mice, which may be further expanded by treatment with DMXAA, resulting in a significant enhancement of E7-specific CD8+ immune responses in treated mice (Figure 2).

For clinical translation, it is important to determine the optimal regimen for treatment with DMXAA. Our study showed that administration of DMXAA 3 days after the first CRT/E7 DNA vaccination generates the best antigen-specific CD8+ T cell immune responses in vaccinated mice (Figure 3). Our data also indicated that administration of two doses of DMXAA after the first CRT/E7 DNA vaccination generates E7-specific CD8+ T cell immune responses in vaccinated mice (see Additional File 1; Figure S1). Thus, it will be of importance to further explore the optimal treatment for administration of DMXAA in clinical trials.

Our study explored the mechanism of enhancement induced by DMXAA. We found that DMXAA administered after the first DNA vaccination influences the cytokine profile in the serum of mice with observed immune enhancement (Figure 7). Mice treated with DMXAAA after the first DNA vaccination showed upregulation of the cytokines IL-6, G-CSF, KC, MIP-1β and RANTES. IL-6 can be secreted by T cells and macrophages to stimulate immune response to trauma, leading to inflammation (for review see [21]). G-CSF is a cytokine produced by a number of different tissues to stimulate the bone marrow to produce granulocytes and stem cells. KC, MIP-1β and RANTES are chemokines that act as chemo-attractants to guide the migration of T cells. All these molecules are believed to play a role in the immune enhancement generated by DMXAA administration. In additon, our data suggest that treatment with DMXAA 3 days after the first DNA vaccination can lead to enhancement of antigen-specific CD4+ T cells (Figure 5). Thus, it is possible that the enhancement of E7-specific CD8+ T cell responses by DMXAA treatment may also be contributed by both cytokines as well as antigen-specific CD4+ T cells.

Our data also suggested that iNOS plays a role in the immune suppression caused by DMXAA administration at the time of the first DNA vaccination (Figure 8). Our study also showed that the immune suppression mediated by DMXAA is abolished in iNOS knockout mice. Because DCs are essential for priming of antigen-specific CD8+ T cell immune response, it is conceivable that treatment with DMXAA may lead to the negative impact on DC function, presumably mediated by iNOS. It will be of interest to further characterize the role of iNOS on immunosuppression mediated by DMXAA treatment.

In summary, we have demonstrated that the combination of DMXAA treatment with HPV-16 E7 DNA vaccination can enhance or suppress the antitumor effects and E7-specific CD8+ T cell immune responses in treated mice depending on the time of administration of DMXAA. These results may have potential implications for future clinical translation.

## Competing interests

The authors declare that they have no competing interests.

## Authors' contributions

SP was involved in the execution of the project. AM was involved in the interpretation of the data and writing the manuscript. XP participated in the design of the study and the statistical analysis. CFH and TCW provided overall supervision and guidance for the project. All authors read and approved the manuscript.

## Supplementary Material

Additional File 1 **Figure S1. Characterization of DC number and function**. 5-8 week-old C57BL/6 mice (3 mice/group) were injected with 1 × 10^5 ^TC-1 cells subcutaneously. On day 13 after tumor injection, the mice were vaccinated with 2 μg of pcDNA3-CRT/E7 via gene gun delivery and boosted 3 days later. 3 days after the first vaccination, mice was treated with 20 mg/kg DMXAA intraperitoneally, and another group was given same volume of vehicle (5% NaHCO3). 24 hours later, the tumor draining lymph nodes were harvested and single cell preparation was prepared. The cells were then stained with anti-mouse CD45-FITC, anti-mouse CD11c-APC, plus one of the following PE-conjugated antibodies: anti-mouse ICAM-1, CD40, CD80, CD86. The cells were gated on CD45 and CD11c positive population. **(A) **Representative flow cytometry data. **(B) **Bar graph representing the expression of DC activation markers. The number in the figure represents mean fluorescence intensity (MFI). **(C) **Bar graph representing the percentage of CD11c+CD45+ DCs. ** indicated p < 0.001. Click here for file

Additional File 2 **Figure S2. Characterization of the E7-specific CD8+ T cell immune responses in mice treated with HPV16 E7 DNA vaccine in combination with two doses of DMXAA**. **(A) **Schematic diagram of the immunization regimen of the CRT/E7 DNA vaccine and DMXAA. 5-8 weeks old C57BL/6 mice were vaccinated with pcDNA3-CRT/E7 DNA vaccine via gene gun delivery and treated with either one dose or two doses of DMXAA as indicated in **Figure 6A**. One week after last vaccination, splenocytes from mice were harvested and characterized for E7-specific CD8^+ ^T cells using intracellular IFN-*γ *staining followed by flow cytometry analysis. **(B) **Representative data of intracellular cytokine staining followed by flow cytometry analysis showing the number of E7-specific IFNγ+ CD8+ T cells after DMXAA treatment. (C) Bar graph depicting the number of E7-specific IFNγ+ CD8+ T cells per 3'10^5 ^splenocytes ± SEM following DNA vaccination +/- DMXAA treatment. The data shown here are from one representative experiment of two performed. Click here for file

Additional File 3 **Figure S3. Characterization of the apoptotic T cell death induced by DMXAA**. Bar graph depicting the percentage of annexin V + cells in T cells treated with or without DMXAA. 5-8 weeks old C57BL/6 mice were treated with DMXAA at 20 mg/kg via i.p. injection. 48 hours later, splenocytes were harvested and apoptosis of CD4+ and CD8+ T cells were analyzed by annexin V staining. The data shown here are from one representative experiment of two performed. Click here for file
