# Supplementary figures and images for: Vascular disrupting agent DMXAA enhances the antitumor effects generated by therapeutic HPV DNA vaccines
Source: J Biomed Sci. 2011 Mar 8;18(1):21. doi: 10.1186/1423-0127-18-21 (PMC3062584; doi:10.1186/1423-0127-18-21)

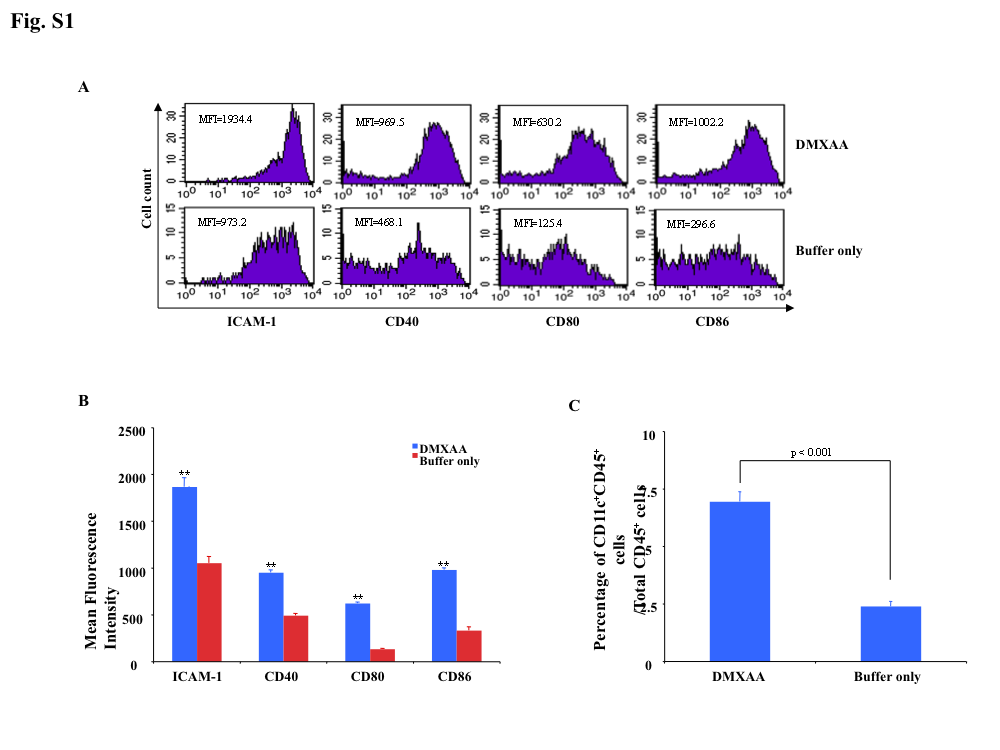

Supplement: Additional File 1 — Figure S1. Characterization of DC number and function. 5-8 week-old C57BL/6 mice (3 mice/group) were injected with 1 × 10^5^ TC-1 cells subcutaneously. On day 13 after tumor injection, the mice were vaccinated with 2 μg of pcDNA3-CRT/E7 via gene gun delivery and boosted 3 days later. 3 days after the first vaccination, mice was treated with 20 mg/kg DMXAA intraperitoneally, and another group was given same volume of vehicle (5% NaHCO3). 24 hours later, the tumor draining lymph nodes were harvested and single cell preparation was prepared. The cells were then stained with anti-mouse CD45-FITC, anti-mouse CD11c-APC, plus one of the following PE-conjugated antibodies: anti-mouse ICAM-1, CD40, CD80, CD86. The cells were gated on CD45 and CD11c positive population. (A) Representative flow cytometry data. (B) Bar graph representing the expression of DC activation markers. The number in the figure represents mean fluorescence intensity (MFI). (C) Bar graph representing the percentage of CD11c+CD45+ DCs. ** indicated p < 0.001. [file 1423-0127-18-21-S1.TIFF]

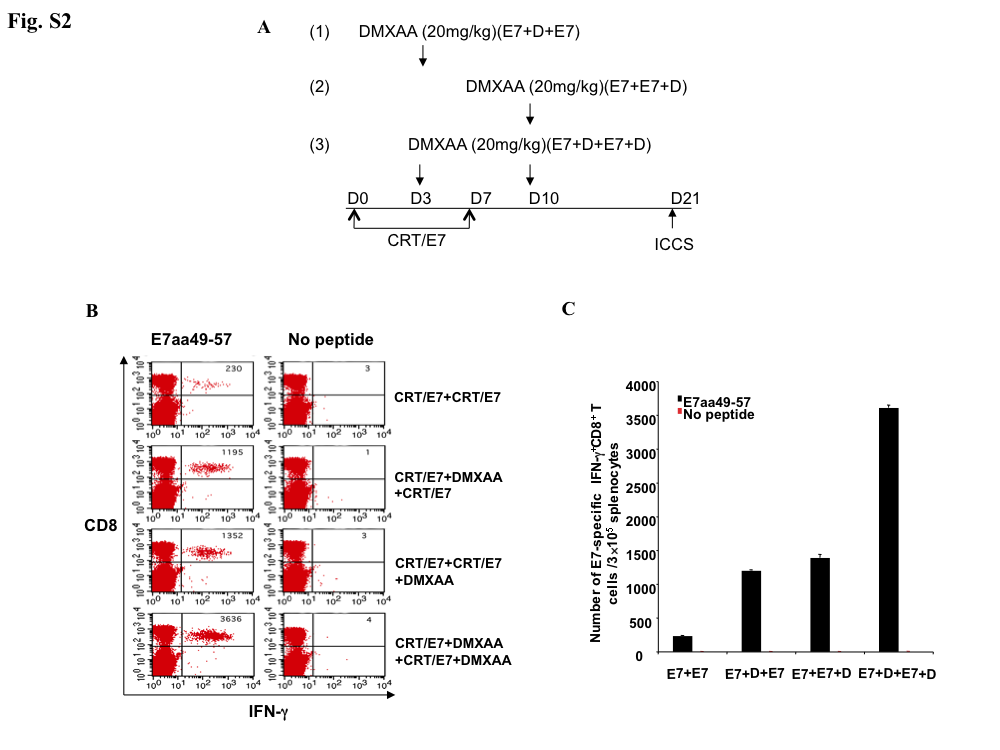

Supplement: Additional File 2 — Figure S2. Characterization of the E7-specific CD8+ T cell immune responses in mice treated with HPV16 E7 DNA vaccine in combination with two doses of DMXAA. (A) Schematic diagram of the immunization regimen of the CRT/E7 DNA vaccine and DMXAA. 5-8 weeks old C57BL/6 mice were vaccinated with pcDNA3-CRT/E7 DNA vaccine via gene gun delivery and treated with either one dose or two doses of DMXAA as indicated in Figure 6A. One week after last vaccination, splenocytes from mice were harvested and characterized for E7-specific CD8+ T cells using intracellular IFN-γ staining followed by flow cytometry analysis. (B) Representative data of intracellular cytokine staining followed by flow cytometry analysis showing the number of E7-specific IFNγ+ CD8+ T cells after DMXAA treatment. (C) Bar graph depicting the number of E7-specific IFNγ+ CD8+ T cells per 3'105 splenocytes ± SEM following DNA vaccination +/- DMXAA treatment. The data shown here are from one representative experiment of two performed. [file 1423-0127-18-21-S2.TIFF]

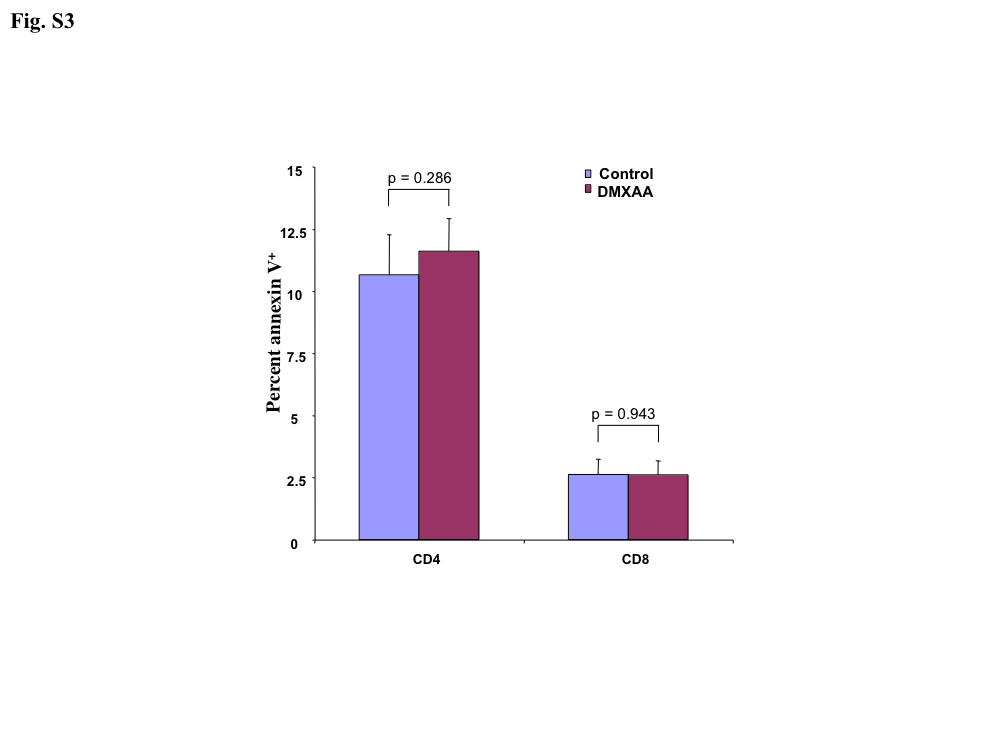

Supplement: Additional File 3 — Figure S3. Characterization of the apoptotic T cell death induced by DMXAA. Bar graph depicting the percentage of annexin V + cells in T cells treated with or without DMXAA. 5-8 weeks old C57BL/6 mice were treated with DMXAA at 20 mg/kg via i.p. injection. 48 hours later, splenocytes were harvested and apoptosis of CD4+ and CD8+ T cells were analyzed by annexin V staining. The data shown here are from one representative experiment of two performed. [file 1423-0127-18-21-S3.TIFF]
